# Dual Modification of Sago Starch via Heat Moisture Treatment and Octenyl Succinylation to Improve Starch Hydrophobicity

**DOI:** 10.3390/polym14061086

**Published:** 2022-03-08

**Authors:** Angela Myrra Puspita Dewi, Umar Santoso, Yudi Pranoto, Djagal W. Marseno

**Affiliations:** 1 Department of Food and Agricultural Product Technology, Faculty of Agricultural Technology, Gadjah Mada University, Yogyakarta 55281, Indonesia; a.puspita@unipa.ac.id (A.M.P.D.); umar_s@ugm.ac.id (U.S.); pranoto@ugm.ac.id (Y.P.); 2 Department of Agricultural Technology, Faculty of Agricultural Technology, Papua University, Manokwari 98314, Indonesia

**Keywords:** sago starch, heat moisture treatment, octenyl succinic anhydride

## Abstract

To elucidate the pretreatment of a heat moisture treatment that could increase the DS and hydrophobicity of OSA starch, the effect of the moisture level of the HMT process on the physicochemical properties was investigated. The higher moisture content (MC) in the HMT process led to a decreasing degree of crystallinity and gelatinization enthalpy and also produced surface damage and cracking of the granules. HMT pretreatment with the right moisture content resulted in OSA starch with the maximum DS value and reaction efficiency. Pre-treatment HMT at 25% MC (HMT-25) followed by OSA esterification exhibited the highest DS value (0.0086) and reaction efficiency (35.86%). H25-OSA starch has been shown to have good water resistance (OAC 1.03%, WVP 4.92 × 10^−5^ g/s m Pa, water contact angle 88.43°), and conversely, has a high cold water solubility (8.44%). Based on FTIR, there were two new peaks at 1729 and 1568 cm^−1^ of the HMT-OSA starch, which proved that the hydroxyl group of the HMT starch molecule had been substituted with the carbonyl and carboxyl ester groups of OSA.

## 1. Introduction

Starch is a carbohydrate source that is widely used for food and non-food applications. One source of starch that has high potential to be used in various food and non-food industries is sago (*Metroxylon* sp.) because of its abundance, low cost, it can easily be gelatinized, has high viscosity, is easy to form, and low syneresis [1,2]. However, sago starch has hydrophilic properties that are limited in applications of various products such as biodegradable films, so modifications are needed to reduce its hydrophilic properties. Several studies have been carried out to reduce the hydrophilicity of starch-based biodegradable films by chemical modification through hydroxypropylation [3], oxidation [4,5], and octenylsuccination [6,7]. The amount of literature in the field of Octenyl succinic anhydride (OSA) starch synthesis and characterization have increased significantly in the last decade. Octenyl succinic anhydride (OSA) is a commonly used esterification agent for modifying starches and has been permitted for food application since 1972 [8,9]. Starch modified with OSA has been approved for food use by the Food and Drug Administration (FDA) with a limit of 3% of OSA [10]. Esterification of starch with OSA involves the partial substitution of a hydroxyl group with a hydrophobic substituent, thereby giving its amphiphilic character and interfacial properties [11]. 

OSA modifications in several types of starch sources have been reported in several scientific literatures such as sago starch [12,13], Indica rice starch [14], pearl millet starch [15], cassava starch [9], waxy corn starch [16], potato starch [11,17], and sweet potato starch [6,18]. Several methods of OSA-starch synthesis have been reported, but starch modification with OSA in aqueous media remains the most widely used because it does not require unsafe organic solvents and permits appropriate response results under slight experimental conditions (temperature around 30–35 °C and pH around 8.5) [19]. Some changes in physicochemical properties caused by OSA modification include a decrease in temperature and enthalpy of gelatinization, an increase in swelling power, paste viscosity and paste clarity, and decreased digestibility. These changes are influenced by the degree of substitution (DS) and reaction efficiency (RE). Generally, the reaction between OSA and starch is inhibited due to the low solubility of OSA in water and uneven poor penetration of large OSA oil droplets into starch granules in an aqueous suspension [11,13]. Therefore, it has limited access to the interior of semi-crystalline starch granules. Several attempts have been made to increase the DS value and reaction efficiency by destroying the compact structure of starch granules, increasing the surface area, reducing crystallinity, reducing OSA drop size, and/or dispersing starch molecules, thus enhancing OSA accessibility to hydroxyl groups and a more homogeneous distribution of OS groups in the final product [9,14,20]. 

Heat moisture treatment (HMT) is a physical modification that involves incubating starch granules at a moisture content of <35% *w*/*w* for a certain period at temperatures above glass temperature (Tg) but below the gelatinization temperature [21]. HMT has been shown to decrease relative crystallinity, rearrangement of amylopectin chains, and cause changes in starch granule morphology [22,23,24]. These changes are influenced by the effect of moisture level, length of heating time, and heating temperature during the HMT process. Several studies have reported that pre-treatment of HMT before OSA esterification could increase the entry of OSA into starch granules and increase the reaction between OSA and the hydroxyl groups of starch increasing DS [13]. 

Previous studies have reported that HMT before OSA modification increased DS and reaction efficiency of sweet potato, cassava, and corn OSA-starches by the reduction in starch crystallinity and the formation of channels, voids, and hollows inside the granules, which facilitate the entry and reaction of OSA inside the granules [9,18,25]. However, few studies have reported the effect of changes in the physicochemical properties of HMT sago starch on the degree of substitution, reaction efficiency, and hydrophobic properties of OSA modified sago starch. Therefore, the purpose of this work was to investigate the impact of the changes in the physicochemical properties of HMT-modified sago starch on the DS value, reaction efficiency, and hydrophobicity properties of OSA-modified sago starch.

## 2. Materials and Methods

### 2.1. Materials

Sago starch (*Metroxylon* sp.) was obtained from the local market in Manokwari, West Papua, Indonesia. The starch was washed and filtered three times to remove impurities and then dried in a cabinet dryer at 50 °C for 24 h. Dried sago starch was ground with a mortar and pestle then sieved through a 0.149 mm sieve and then stored in an airtight container until further use. 2-Octen-1-ylsuccinic anhydride (OSA) was purchased from Sigma-Aldrich Corporation (St. Louis, MO, USA), and all the reagents used in this study were of analytical grade.

### 2.2. Starch Modification

#### 2.2.1. Heat Moisture Treatment (HMT) Modified Sago Starch

Sago starch with an initial moisture content determined was adjusted to the moisture content treatment of 10%, 15%, 20%, 25%, and 30% (HMT 10, HMT 15, HMT 20, HMT 25, and HMT 30, respectively) by spraying the calculated amount of distilled water onto the starch and then homogenized for 20 min. The exact moisture content of the mixture was measured with a moisture analyzer (MB120, Ohaus, Shanghai, China). The moist starch was then placed in a 500 mL Duran glass bottle equipped with a screw cap and incubated for 1 h before being placed in an autoclave and heated for 1 h at 120 °C [26]. The glass bottle was left to cool to room temperature, then the starch was dried at 50 °C in a cabinet dryer. HMT starches with the lowest crystallinity degree and enthalpy value continued to OSA modification.

#### 2.2.2. OSA Modified Sago Starch

The preparation of OSA starch followed the method of Jiranuntakul et al. [9]. Native and selected HMT sago starches were dispersed in distilled water (30%) and then the pH of the suspensions was adjusted to 8.5 with a pH meter by adding 3% NaOH solution. A sample of 3% OSA (based on dry starch basis) was slowly added (diluted five times with isopropyl alcohol, *v*/*v*) within a period of 2 h while maintaining the pH and constant stirring. Esterification was conducted for 4 h at 35 °C, and then the pH was adjusted to 6.5 with 3% HCl solution at the end of the reaction. The OSA starch was collected by vacuum filter, washed two times with distilled water, two times with 70% aqueous alcohol, and then dried at 40 °C for 24 h.

### 2.3. Physicochemical Properties of Native and Modified Sago Starch Determination

Swelling power, solubility, water absorption capacity, and amylose contents were determined to characterize the physicochemical properties of native, HMT, and HMT-OSA starch. The swelling power and solubility of the starches were determined following the method of Zavareze et al. [27]. To determine the water/oil absorption capacity of starches, 10 mL of distilled water/soybean oil were used following the method by Uzomah and Ibe [28]. Amylose content was determined using the method by Bharti et al. [22] with slight modification. The 100 mg sample was added to 1 mL 95% ethanol and 9 mL of 1 N NaOH in a tube. The sample was heated at 90 °C for 30 min, cooled down, and transferred into a volumetric flask, where the volume was made up to 100 mL using distilled water. Then, 5 mL was put into a 100 mL volumetric flask with 1 mL of 1 M acetic acid and 2 mL iodine solution and diluted to 100 mL. The solution was incubated for 20 min and the absorbance was measured at 640 nm by spectrophotometer (Genesys 10S, UV–Vis spectrophotometer, Thermo Scientific, Madison, WI, USA). Amylose content was calculated against a standard curve prepared using amylose (from potato) (Sigma Aldrich, Taufkirchen, Germany, purity ≥ 98%).

### 2.4. Pasting Properties Analysis

The pasting property of native and HMT starch samples was evaluated using a Rapid Visco Analyzer (RVA-4500, Perten Instruments, Waltham, MA, USA). The starch sample (3.5 g, dry basis) was added to 25 mL of distilled water in the RVA canister. The sample was held at 50 °C for 1 min, then the starch suspension was heated from 50 °C to 95 °C at a rate of 5.2 °C/min, held at 95 °C for 5 min, cooled to 50 °C at 5.2 °C/min, and held at 50 °C for 2 min. The paddle speed was 960 rpm for the first 10 s, and 160 rpm for the rest of the experiment. Parameters including pasting temperature, peak viscosity, trough viscosity, final viscosity, breakdown viscosity, and setback viscosity were recorded.

### 2.5. Paste Clarity Determination

Paste clarity of starch solutions determined following the method of Mehboob et al. [29]. A sample of 1% (*w*/*v*) of the starch slurry was heated in a boiling water bath for 30 min with intermittent mixing, then cooled to room temperature. The light transmittance (%T) was measured at 650 nm against distilled water as the blank using a UV–Visible Spectrophotometer (Genesys 10S, UV–Vis spectrophotometer, Thermo Scientific, Madison, WI, USA). The samples were stored at 4 °C and percent transmittance (%T) was measured at 0 h, 24 h, 72 h, and 168 h.

### 2.6. Analysis of the Thermal Properties 

Thermal characteristics of the native and modified starches were determined using a differential scanning calorimeter (DSC-60 Plus, Shimadzu, Ikenohata, Taito-ku, Tokyo, Japan). Starch (3.5 mg, dry weight) was loaded into an aluminum pan and 5 µL of distilled water was added using a micro-syringe. Samples were hermetically sealed and allowed to stand for 1 h at room temperature before heating in the DSC. Sample pans were heated at a rate of 10 °C/min from 30 to 110 °C and an empty pan was used as the reference.

### 2.7. X-ray Diffraction Analysis

The X-ray patterns were obtained using an X-ray diffractometer (Bruker D2, Phaser, Germany) with Cu Kα radiation (λ = 0.154 nm). The scanning region of the diffraction angle (2θ) was from 4°–35° at 25 °C. The total area under the curve and the area under each prominent peak (crystalline area) were determined using Origin Pro software (OriginPro 2019, OriginLab Corporation, Northampton, MA, USA) [30]. The total relative crystallinity (RC) of the samples was calculated as follows:

RC (%) = (*Ac*/*At*) × 100

where *Ac* is the area of the crystalline peak and *At* is the total area under the curve.

### 2.8. Morphology Granules Analysis

The surface morphology of native and modified starch granules was examined using scanning electron microscopy (JSM-6510LA, JEOL, Akishima, Tokyo, Japan). Native and modified starch samples were mounted individually on the stubs using double-sided adhesive tape and the starch samples were coated with gold. Micrographs were taken at 500× magnification and 5 kV accelerating potential.

### 2.9. Degree of Substitution and Reaction Efficiency Determination

The determination of the degree of substitution (DS) was based on the study by Bhosale and Singhal [16] by alkali saponification followed by back titration of excess alkali using the titrimetric method. A blank titration was also performed by using native starch. DS was calculated as follows:

$$\mathrm{OSA}\mathrm{substitution}\;(\%)=\frac{\left(Vb-Vs\right)\times 0.1\times N\times 100\;}{W}$$

where *Vb* is the volume of HCl required for blank titration; *Vs* is the volume of HCl required to titrate the sample; *W* is the weight (g) of sample; and *N* is the normality of the HCl solution

$$\mathrm{DS}=\frac{162\times \%\;OSA\;substitution}{21000-\left(209\times \%\;OSA\;substitution\right)}\;$$

where 162 is the molecular weight of the glucose unit; 21,000 is the 100 × molecular weight of octenyl succinyl group; and 209 is the molecular weight of octenyl succinyl group minus the molecular weight of hydrogen atom

The reaction efficiency (*RE*) was calculated as follows:

$$\mathrm{RE}=\frac{actual\;DS}{theoretical\;DS}\times 100\%$$

where *Theoretically DS* is calculated by assuming that all the added OSA reacted with starch to form the ester derivative.

### 2.10. Water Contact Angle Measurement

**Figure 1:**
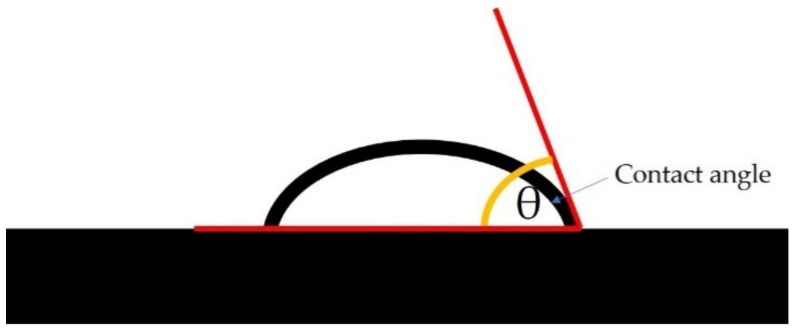
Schematic diagram demonstrating the contact angle measurement.

Water contact angles were analyzed using a custom-built optical sessile drop system equipped with a USB Digital Microscope [31,32]. Native and modified starch samples were prepared through solution casting. The starch samples (3%) were dispersed in distilled water and heated at 90 °C, then the suspensions were poured onto glass dishes, and dried at 40 °C for 10 h. About 3 μL of a deionized water droplet was dropped onto the surface of the samples, and images were captured using a video camera. These images were taken at 0, 45, and 90 s after dropping the water. WCAs were analyzed using ImageJ software by calculating the tangent of the liquid-solid interface and the liquid-vapor interface intersection (Figure 1). The reported result is the average value of at least five different measurements.

### 2.11. Water Solubility Test

The water solubility of native and modified starch followed the method described by Fang et al. [33]. A total of 10 g of the starch samples were suspended in 100 g of distilled water. Starch suspensions were stirred at room temperature (27 °C) for 30 min and then centrifuged at 1000 rpm for 15 min. The supernatants were poured into a pre-weighed Bekker glass and dried at 105 °C for 8 h. The weight of the dried supernatant was measured until constant weight and water solubility was calculated as follows

$$\mathrm{Water}\mathrm{Solubility}\;(\%)=\frac{weight\;of\;dissolved\;solids\;in\;supernatant}{weight\;of\;sample}\;\times \;100\%$$

### 2.12. Water Vapor Permeability Test

Water vapor permeability (WVP) of the native and modified starch was measured according to the ASTM E96-92 method [34]. Native and modified starch samples were prepared as the method of determining the contact angle measurement. A sample was placed in between the cup and the ring cover of the acrylic cup filled with 20 g of silica gel (RH ≈ 0%). Circular acrylic cups containing the material were placed in desiccators at a relative humidity (RH) of ∼60% and room temperature. Weight gain measurements were taken by weighing the acrylic cup every 3 h for 72 h. The WVP was calculated as follows:

$$\mathrm{WVP}\;(g/s\;m\;\mathrm{Pa})=\frac{a\times d}{Po}$$

where *a* is the slope of plot weight gain versus time; *d* is the thickness of the samples; and *Po* is the saturation vapor pressure of water at room temperature.

### 2.13. Fourier Transform Infrared Spectroscopy (FTIR) Analysis

FTIR spectra of native and modified starches were determined using a FTIR spectrophotometer (FTIR Thermo Nicolet IS 10, Thermo Fischer Scientific, Madison WI, USA). Before the preparation of the pellet, the starch samples were dried at 105 °C for 12 h to remove any moisture. To make a thin pellet, the dried sample was finely ground with KBr in a ratio of 1:100 (*w*/*w*) and scanned in the wavenumber range of 400–4000 cm^−1^ with a resolution of 8 cm^−1^.

## 3. Results

### 3.1. Physicochemical Properties

Effects of moisture treatment of the HMT process on HMT sago starches are shown in Table 1. In determining the swelling power and solubility, starch granules swell and amylose-amylopectin will spread out from the granules. Sago starch has a relatively low swelling power ranging from 18.59 g/g–21.01 g/g, while the solubility of sago starch is relatively high, ranging from 36.10–38.63% [35]. From Table 1, the swelling power and solubility of native starch were higher than that of the HMT starches. Several researchers have also reported that forage decreased the swelling power and solubility of sago and arenga starch [36], sweet potato [37], and rice starch [27]. HMT affected the rearrangement of starch granules and increased the interaction of amylose–amylose and amylose–amylopectin chains, thereby reducing the swelling power. During the HMT process, the mobility of molecules increases, resulting from the interaction of amylose–amylose and amylose–amylopectin chains, which decreases the available hydroxyl groups to hydration and the diffusion of amylose–amylopectin molecules [23]. Reduction in the solubility of HMT starches due to the rearrangement of starch granules following interaction between amylose–amylopectin molecules and the formation of double helices in the side chain of amylopectin, preventing them from leaching out of the granules [8,19,29].

Water absorption capacity is the ability of starch to bind water against gravity. The ability to bind water is influenced by the number of water-binding sites, the type of modification, and the extent of severity [38]. Table 1 shows that the WAC of HMT starches treated with a moisture level of 25% and 30% was higher (1.80 and 1.93 g/g) than that of native starch (0.89 g/g), while the WAC of HMT starches treated with a moisture level of 20% was not significantly different than that of native starch. Similar results were previously reported in African Yam bean starch, where WAC starch increased after hydrothermal treatment and a higher moisture level of heat moisture treatment due to an escalation in the hydrophilic tendency of starch [39]. HMT resulted in a reduction in starch crystallites, which increased the hydrophilic affinity, thereby increasing the water-binding capacity [22].

The amylose content of starch was classified into low amylose content (<20%), medium amylose content (20–25%), and high amylose content (>25%) [40]. The amylose content of sago starch from Papua, Indonesia is relatively high, ranging from 33.81–38.47%, depending on the variety of sago palms [41]. From the results of the study, the amylose content of native sago starch was 36.18%, which was similar to the results of previous studies (Table 1). HMT modification had no effect on starch amylose content. Previous studies have also reported that HMT modification did not change the amylose content in chestnut starch [42], mango kernel starch [22], and sorghum starch [43].

### 3.2. Pasting Properties

**Figure 2:**
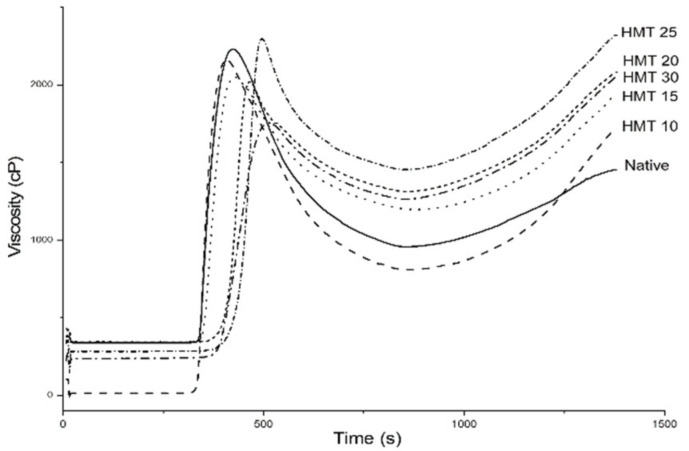
Pasting properties of native and HMT sago starch samples. Native indicates native starch, HMT indicates heat-moisture treatment starches. HMT 10, HMT 15, HMT 20, HMT 25, and HMT 30 indicate the starch samples treated with HMT for the 10%, 15%, 20%, 25%, and 30% moisture treatments, respectively.

The pasting properties of native and HMT sago starches are shown in Figure 2. After being modified by HMT, the pasting parameters changed. Moisture level escalation of HMT treatment caused the peak, trough, and breakdown viscosity to decrease, while the final viscosity, setback, pasting temperature, and peak time increased. The drastic difference in pasting properties due to HMT modification has also been reported in bean starch [44]. The decrease in peak, trough, and breakdown viscosity was due to the inhibition of HMT starch to swell (Table 1), while the increase in setback was associated with a decrease in HMT starch solubility (Table 1). Modification of HMT pasting temperature, along with the higher water content treatment, obtained the highest pasting temperature from HMT25 treatment (25% moisture content) at 80.95 °C while the pasting temperature of native starch was 75.05 °C. The pasting temperature of natural sago starch obtained had the same value as reported in previous studies, which ranged from 74.5–75.80 °C [41]. The increase in the pasting temperature of the HMT starch occurred due to the reorientation of the starch granules [43].

### 3.3. Paste Clarity

**Figure 3:**
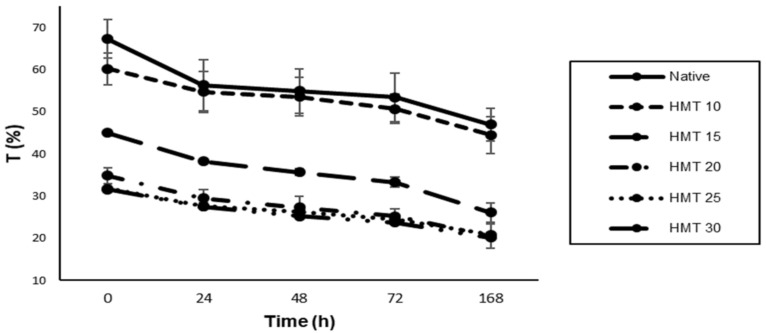
Paste clarity of native and HMT sago starch samples. Native indicates native starch, HMT indicates heat-moisture treatment starches. HMT 10, HMT 15, HMT 20, HMT 25, HMT 30 indicate the starch samples treated with HMT for the 10%, 15%, 20%, 25%, and 30% moisture treatments, respectively.

The paste clarity of native starch and HMT starch during storage was presented in Figure 3. Overall paste clarity of HMT starches was lower compared to native starch (*p* < 0.05). Reduction in the light transmission in HMT starch might be due to increased flexibility of the starch chain in the amorphous region and amylose leaching during pasting [42]. During storage under refrigerated conditions, the paste clarity of native and HMT starches was decreased. A similar trend in the reduction in the paste clarity of HMT starches during cold storage has been reported in mango kernel starch [22]. The increase in starch paste opacity during storage was associated with leached amylose and amylopectin chains, granular remnants, and granular swelling. 

### 3.4. Thermal Properties

**Figure 4:**
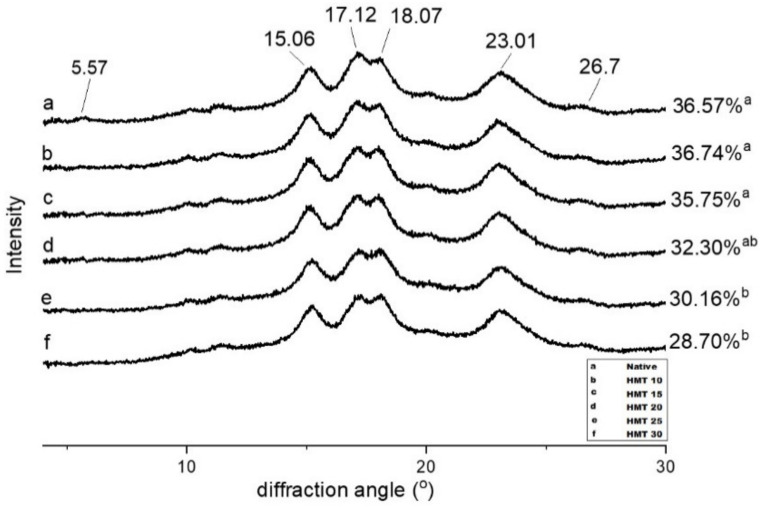
X-ray diffraction patterns and relative crystallinity of the native and HMT sago starch samples. (**a**–**f**) indicate native, HMT 10, HMT 15, HMT 20, HMT 25, and HMT 30, respectively.

Thermal properties of native and HMT sago starches were analyzed using DSC (Table 2). Modification of HMT altered the thermal properties of sago starch by increasing the moisture levels while the HMT process promoted an increase in the thermal stability of starches by bringing the onset (To), peak (Tp), and final (Tc) gelatinization temperatures to higher values, while the gelatinization temperature range (Tc–To) and enthalpy gelatinization decreased. The results were in agreement with those reported for buckwheat starch [45,46], sago and arenga starch [47], and glutinous rice starch [24]. The increase in To, Tp, and Tc of HMT starches was related to the strengthening of interactions between amylose and amylopectin branching, suppressing starch chain movement in amorphous regions, and promoting the diffusion of starch molecules. As a result, starch modified by this physical treatment requires a higher temperature to achieve swelling, which in turn disrupts the starch crystal domains and causes an increase in the transition temperature [24,48]. Gelatinization range (To–Tc) was indicated as the heterogeneity of crystals in starch granules. A low To–Tc exhibited crystal perfection, while a high value represented a fusion of crystals with different stability [49]. The (Tc–To) of the HMT sago starches decreased with increasing moisture level from 20% to 30% of the treatments compared to native starch, indicating a greater homogeneity of the crystallites. In this study, the HMT starches displayed lower enthalpy (∆H) compared to native starch, indicating disruption of the double helices in the crystalline and non-crystalline regions, which is consistent with the relative crystallinity data from XRD (Figure 4).

### 3.5. X-ray Diffraction Pattern and Relative Crystallinity

XRD pattern and relative crystallinity of native and HMT sago starches are shown in Figure 4. The diffraction pattern of sago starch and HMT starches showed type C crystals (a combination of diffraction types A and B) with strong peaks of 2θ at around 5.57°, 15.06°, 17.12°, 18.07°, 23.01°, and 26.7°, with a relative crystallinity of native sago starch of 36.56%, as reported in previous studies [36,41]. The peak at a 2θ value of 17° is a feature of the B-type crystal, while the peak at 23° is characteristic of the A-type crystal [50]. After HMT modification, the diffraction pattern did not change compared to native starch. However, the peak intensity at a 2θ value of 5.57°, 17.12°, and 18.07° decreased with elevated moisture level treatment (Supplementary Materials Table S1). Similar results have also been reported in sweet potato starch [51], buckwheat starch [45], and rice starch [27]. Overall, the relative crystallinity data were inversely proportional to moisture level treatment. This phenomenon has also been reported in potato starch [52], rice starch [24,27], and mango kernel starch [22]. Excess water during the HMT process accompanied by high-temperature heating caused hydrogen bond breakdown, movement, and dissociation of double helices, which is associated with a reduction in crystalline order during HMT [24,53]. Changes in the crystallinity of HMT starches might explain the greater enzymatic susceptibility and enhanced reaction to chemical modification, as evidenced by the heat-moisture treatment [9,54].

### 3.6. Granule Morphology

**Figure 5:**
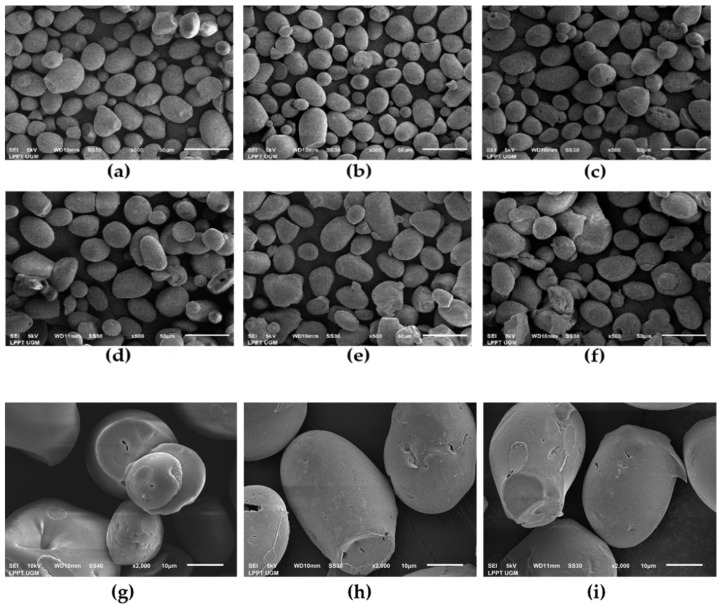
Scanning electron microscopy (SEM) images of native, HMT, and OSA starch samples. (**a**) = native starch; (**b**) = HMT 10; (**c**) = HMT 15; (**d**) HMT 20; (**e**) = HMT 25; (**f**) = HMT 30 at magnification images of 500×; (**g**) = HMT25; (**h**) = Native-OSA starch; and (**i**) = HMT25-OSA at magnification images of 2000×.

The morphology and granule structure of the native sago starch and HMT starches were observed using SEM and are presented in Figure 5. The shape of the native sago starch granules was mainly oval with a smooth surface, indicating that the starch did not change due to the extraction process with an average granule size of 29.22 µm, which is in agreement with previous studies [35,41,55]. In this study, there was no change in the shape of the sago starch granules after HMT at 10%, 15%, 20%, and 25%, but there were roughness, cracks, and hollows in the center of the granules while an agglomeration of starch granules formed with treatment at 30% MC (HMT-30). These cavities and holes were caused by the recombination of amylose and amylopectin chains, which contributed to a more compact amorphous region [45]. The rough surface was due to the evaporation of water molecules and induced the chain of amylopectin double helices to rearrange into a denser packaging structure that would act as a barrier to water penetration into the starch granules, thereby shifting the gelatinization endothermic to a higher temperature [56]. The intensity of these changes was moisture level-dependent (Figure 4d–f). This is in agreement with several previous studies where the high moisture level of the HMT process caused partial gelatinization and agglomeration [22,23,27,45].

### 3.7. Degree of Substitution and Reaction Efficiency

Based on the crystallinity and enthalpy data of HMT starches (Figure 3 and Table 2), HMT starches were selected from the moisture level treatments of 20% (HMT20), 25% (HMT25), and 30% (HMT30) for further OSA modification. The degree of substitution and the reaction efficiency of HMT-OSA starch is shown in Table 3. The DS and RE values of HMT-OSA starches were higher compared to native starch (N-OSA), indicating that more hydroxyl groups substituted per glucose unit with OSA in HMT starches. The same results have also been reported from previous studies on cassava starch [9], sweet potato starch [18], and sago starch [13]. The reduction in relative crystallinity as well as the movement of the chain of amylopectin double helices induced by HMT increased the amorphous area, and/or changed the orientation of the crystals, resulting in more sites for reaction. Channels or hollows in the granules generated after HMT are also associated with increasing RE by facilitating the entry of OSA into starch molecules in the granules, which is in agreement with the data of the morphology granules (Figure 4) [9]. 

Moisture level treatment during HMT affected the DS and RE of OSA starch, where the highest DS and RE were obtained from the HMT treatment sample with 25% moisture content treatment (HMT25-OSA). There was a reduction in the DS value and reaction efficiency of H30-OSA, indicating excessive moisture content while the HMT process was not effective in esterification of the OSA reaction. The higher moisture content makes starch granules active due to water absorption throughout treatment, promoting their expansion, providing morphological changes, and also reducing double helices in the crystalline and amorphous regions of starch granules driven by the thermal force, which produced granule agglomeration and crush, consistent with the SEM images, decreasing enthalpy and gelatinization temperature (Table 2), and increasing solubility of HMT-30 (Table 1). (Figure 5) [48]. Tan et al. [53] reported breadfruit starch modified with HMT at 30% and 35% MC produced aggregation of starch granules, moreover, its endothermic peak shrank or even disappeared. The aggregation of starch granules might be suggested to reduce the sites of OSA esterification, thus decreasing the DS value and reaction efficiency.

### 3.8. Water Contact Angle

**Figure 6:**
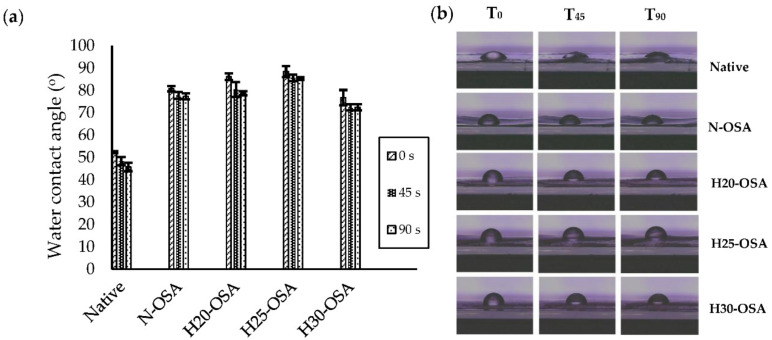
A plot of water contact angle values of native and modified sago starches at varied times (**a**) and contact angle images of native and modified sago starches as a function of time (**b**). N-OSA: native sago starch-OSA modification; H20-OSA: HMT 20-OSA modification; H25-OSA: HMT 25-OSA modification; H30-OSA: HMT 30-OSA modification; T_0_, T_45_, T_90_ represent water contact angles of treated starch samples for 0 s, 45 s, and 90 s, respectively.

Water contact angle measurement enables the qualitative estimation of changes in the hydrophobicity of modified sago starch. The water contact angle values of native sago starch and HMT-OSA sago starch are presented in Figure 5. Native sago starch showed the lowest water contact angle value (52.38°) compared to native-OSA starch and HMT-OSA starches, while the HMT starch was not able to hold water droplets on its surface, thus the water contact angle was not detected. The reduction in the degree of crystallinity, increasing the amorphous region of HMT starch, led to the strong interaction between the water and hydroxyl groups of the starch molecules [57]. The low water contact angle is associated with a high content of hydroxyl groups of glucose units (three hydroxyl groups per part of glucose) [58]. Modified OSA sago starch (N-OSA) showed an increase in hydrophobicity (80.82°) with a higher water contact angle value than native starch counterparts, but its value was still lower than the dual modified starch H20-OSA and H25-OSA. The H25-OSA starch sample showed the highest initial water contact angle (88.43°). Moreover, the data in Figure 6a also showed that the value of the water contact angle decreased with time, which was associated with the spreading of the water drop [59]. It was observed that the water drop spread rapidly in native starch, whereas in HMT-OS starches, the alteration of the water contact angle was relatively slow. The water contact angle of the H25-OSA sample was relatively stable from 0–90 s, suggesting that HMT25 was the best treatment to produce hydrophobic OSA starch, which is associated with DS and RE data in the H25-OS starch sample. In addition, there was an alteration in the surface of the granules, which became rougher after OSA esterification (Figure 5h,i). The roughness of the granule surface could have increased the lipophilic affinity so that it was able to retain water droplets on the surface [60]. Hydrophobization of starch with OSA has the potential to produce a modified starch with higher hydrophobicity than native starch because the hydroxyl groups in the starch molecule are replaced by lipophilic octenyl groups through esterification [19]. 

### 3.9. Oil Absorption Capacity, Water Solubility, and Water Vapor Permeability

The oil absorption capacity of native, HMT 25, OSA, and H25-OSA starches are shown in Table 4. The OAC of heat-treated OSA starch was higher than native and HMT starch, and was even higher than the control (N-OSA) counterparts, indicating that this method allows hydrophobic starch to be obtained. The oil absorption capacity of HMT starch was higher (0.78 g oil/g) compared to native starch (0.65 g oil/g). The high oil absorption capacity of HMT starch is associated with its granular morphology, which has a rough surface and forms cavities (Figure 5g). The surface roughness, granular expansion, and release of the helical structures of sago starch granules after HMT increased the lipophilic affinity [60]. After OSA modification, there was an increment in the oil absorption capacity of both native and HMT starches. Pre-treatment of HMT followed by OSA modification resulted in higher oil absorption capacity (1.01 g oil/g) than that of starch without pre-treatment (0.85 g oil/g). The high value of the oil absorption capacity of starch was associated with the alteration in the starch granule morphology after OSA modification. Based on Figure 5i, some HMT 25 starch granules showed a lot of corrosion and the surface became rougher after OSA esterification, thus increasing the area contact with oil [22]. Meanwhile, disruption of crystallites after HMT modification facilitated the entry and reaction of OSA inside the granules, therefore more starch hydroxyl groups were substituted by OSA groups, which is in agreement with the DS data (Table 3). Esterification with OSA presented long hydrophobic substituent groups into the starch molecule, which impacted the oil absorption capacity [19]. Oil absorption capacity increase with the esterification of starch with OSA is consistent with previous studies [15].

Dual modification by HMT and OSA affected the water solubility of sago starches. Table 4 shows that native starch showed the lowest cold water solubility (4.14%), while HMT and OSA starches exhibited higher water solubility. Native starches have a semi-crystalline form because they comprise a large number of hydrogen bonds, so they are insoluble in water at room temperature [61]. Within the process of hydrothermal treatment, the crystallinity of the starch decreased due to the breaking of inter- and intra-molecular hydrogen bonds, which induced more exposed hydroxyl groups (-OH) to form hydrogen bonds with water, leading to higher cold-water solubility [33]. The introduction of recent functional groups to amylopectin caused a loosening of its structure and facilitated the penetration of water molecules into amylopectin, thereby promoting an increase in water permeation and leaching of more amylopectin from the pellet and an increase in solubility [62,63].

Water vapor permeability of native and modified sago starches is shown in Table 4. Pre-treatment of HMT followed by OSA esterification exhibited the lowest WVP while the WVP value of sago starch esterified by OSA without pre-treatment of HMT showed no significant difference with native starch. Pre-treatment of HMT accompanied by OSA esterification exhibited the lowest WVP, even the WVP value of sago starch esterified by OSA without HMT pre-treatment showed no significant difference with native starch. This phenomenon suggests that the dual modification of HMT-OSA causes the OSA groups to be distributed throughout the starch granules, especially on the surface. The HMT treatment before OSA esterification led to more OSA groups being able to penetrate the internal starch granule, which is consistent with the DS value data (Table 3). Chen et al. [25] reported that hydrothermal treatment (35% moisture content, 48–62 °C) increased the DS value with an increase in heating temperature; in addition, the OS groups seemed to be distributed throughout the OS starch grains, especially on the surface. More OS groups inside the granules led to the enhancement of the hydrophobic nature of starch films by esterification, thereby reducing the availability of hydroxyl groups and inhibiting the access of water into the matrix [6]. From these findings, HMT-OSA starch could be recommended as a biodegradable packaging material because it has good moisture resistance and excessive solubility.

### 3.10. FTIR

**Figure 7:**
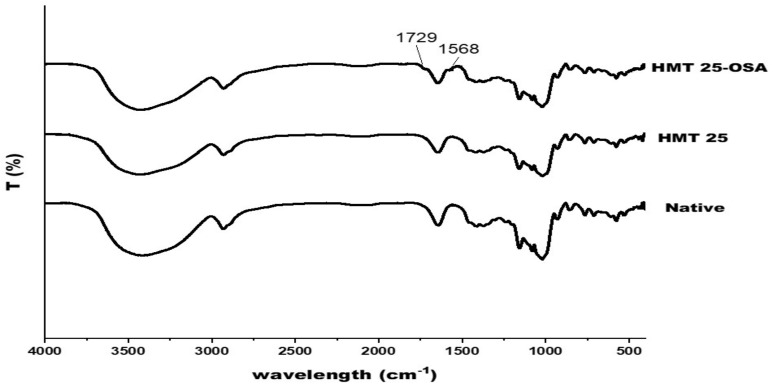
Fourier transform infrared (FTIR) spectrum (wavenumber range 400 and 4000 cm^−1^) of native, HMT, and modified HMT—octenyl succinic anhydride of sago starch. Native: native sago starch; N-OSA: native sago starch—OSA modification; HMT25: HMT sago starch treated 25% moisture treatment; H25-OSA: HMT 25—OSA modification.

**Figure 8:**
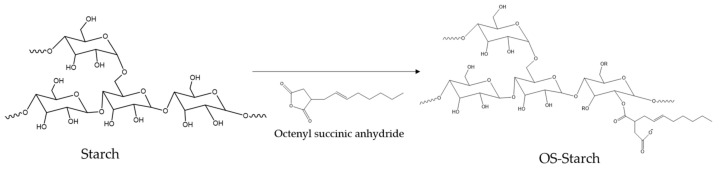
Schematic illustration of the reaction of OSA-starch synthesis.

FTIR was used to verify the structural alteration of the modified sago starches. The comparison of the FTIR spectra of native, HMT 25, and HMT25-OSA starches are shown in Figure 7. Generally, the presence of O–H is shown by the presence of stretching vibration at a wavenumber of approximately 3400 cm^−1^, which is related to intra- and intermolecular hydrogen bonds, while the peak at about 2932 cm^−1^ is associated with –CH_2_ stretching vibration. The peak at the 800–1200 cm^−1^ region of the IR spectrum could be used to analyze the crystalline structure and the short-range order of starch. The absorption bands at 1047 cm^−1^ and 1022 cm^−1^ were associated with the crystalline and amorphous structures of starch, respectively, and the band at 995 cm^−1^ was related to the intramolecular hydrogen bonds of the hydroxyl group at C-6 [64]. The alteration in short-range molecular order of double helices in starch could be measured by the absorbance ratios of 1022/995 cm^−1^ [63]. The HMT starch showed the lowest R_1022/995_ (Table 4), which suggests that the external region was more ordered than the native and OSA starch, respectively, while there was a significant reduction in the short-range order of HMT-OSA, indicating that OS groups were grafted on the surface of the starch granules [65]. Overall, the FTIR spectra of all samples had a similar profile, but there were two new peaks in the HMT25-OSA starch sample at 1729 and 1568 cm^−1^. The peak at 1729 cm^−1^ described a stretch vibration of the C=O ester group, while the peak at 1568 cm^−1^ showed an asymmetrical stretch vibration of the carboxyl group [6,50,51,66]. The appearance of two new peaks in the HMT-OSA starch proved that the hydroxyl group of the starch molecule had been substituted with the carbonyl and carboxyl ester groups of OSA. The results of this study are consistent with previous studies [1,9,67]. Wang et al. [68] reported that the number of OS groups close to the non-reducing ends of the amylopectin molecules elevated with DS. The esterification reaction between starch and OSA occurred at 2-OH, 3-OH, and 6-OH of the glucose residue, and 2-OH possible becomes the main substitution position (Figure 8) [14].

## 4. Conclusions

Heat moisture treatment (HMT) modification affects the physicochemical properties of sago starch where the changes are influenced by the moisture content during the foraging process. HMT caused a reduction in swelling power, solubility, relative crystallinity, gelatinization enthalpy, and paste clarity, but increased WAC, final viscosity, setback, pasting temperature, To, Tp, and Tc gelatinization. The shape of the granules after HMT did not change, but there was roughness, cracks on the surface of the granules, and it was also hollow in the center of the granules with increasing moisture content treatment. The excess moisture content of the HMT process (30% MC) led to agglomeration of the starch granules. Pre-treatment of HMT before OSA esterification increased DS, reaction efficiency, and hydrophobicity of the OSA starch. The combination of HMT25 (25% moisture content) and OSA (H25-OSA) resulted in the highest DS, reaction efficiency, water contact angle, oil absorption capacity, and water barrier. The results obtained from this study suggest that HMT enhances the entry of OSA into starch granules and increases the reaction between OSA and hydroxyl groups of starch via an increased amorphous area; moreover, channels or hollows in the starch granules generated after HMT could facilitate the entry of OSA into starch molecules in the granules.

## Figures and Tables

**Table 1:** Physicochemical properties of the native and HMT sago starches ^†^.

	Swelling Power (g/g)	Solubility (%)	WAC (g/g)	Amylose Content (%)
Native	20.38 ± 0.29 ^a^	38.52 ± 1.10 ^a^	0.90 ± 0.01 ^a^	36.18 ± 0.26 ^a^
HMT 10	17.10 ± 0.05 ^b^	37.87 ± 0.27 ^a^	1.03 ± 0.03 ^a^	35.62 ± 0.58 ^a^
HMT 15	15.24 ± 0.24 ^c^	25.12 ± 1.18 ^b^	1.01 ± 0.05 ^a^	33.76 ± 1.72 ^a^
HMT 20	15.24 ± 0.48 ^c^	25.17 ± 0.77 ^b^	1.12 ± 0.14 ^a^	35.21 ± 0.20 ^a^
HMT 25	15.32 ± 0.36 ^c^	22.49 ± 0.64 ^c^	1.80 ± 0.13 ^b^	36.31 ± 0.36 ^a^
HMT 30	15.71 ± 0.04 ^c^	23.23 ± 0.15 ^bc^	1.93 ± 0.18 ^b^	35.32 ± 0.03 ^a^

^†^ Data are expressed as means ± SD. ^a–c^: Mean values assigned with the same letters within a column are not significantly (*p* < 0.05) different. HMT 10, HMT 15, HMT 20, HMT 25, and HMT 30 represents the samples of sago starch modified by HMT treatments with 10%, 15%, 20%, 25%, and 30% moisture content adjustment, respectively.

**Table 2:** Thermal properties of Native and HMT sago starches ^†^.

	To (°C)	Tp (°C)	Tc (°C)	Tc–To (°C)	∆H (J/g)
Native	72.75 ± 0.03 ^a^	76.70 ± 0.23 ^a^	82.18 ± 2.52 ^a^	9.43 ± 2.55 ^bc^	11.83 ± 0.01 ^a^
HMT 10	71.60 ± 0.13 ^a^	76.14 ± 0.67 ^a^	82.95 ± 0.68 ^a^	11.35 ± 0.81 ^ab^	11.77 ± 1.32 ^a^
HMT 15	73.39 ± 0.29 ^a^	80.73 ± 1.40 ^b^	87.23 ± 2.00 ^b^	13.84 ± 1.71 ^b^	10.09 ± 1.53 ^a^
HMT 20	82.88 ± 1.16 ^b^	87.18 ± 1.21 ^c^	90.06 ± 0.45 ^bc^	7.18 ± 0.71 ^c^	1.88 ± 0.16 ^b^
HMT 25	85.93 ± 1.46 ^c^	90.98 ± 0.21 ^d^	93.18 ± 0.11 ^c^	7.25 ± 1.56 ^c^	1.56 ± 0.43 ^b^
HMT 30	82.68 ± 0.56 ^b^	86.33 ± 0.64 ^c^	88.48 ± 1.56 ^b^	5.80 ± 1.00 ^c^	1.50 ± 0.49 ^b^

^†^ Data are expressed as means ± SD. ^a–d^: Mean values assigned with the same letters within a column are not significantly (*p* < 0.05) different. Native indicates native starch, HMT 10, HMT 15, HMT 20, HMT 25, and HMT 30 indicate the starch samples treated with HMT for the 10%, 15%, 20%, 25%, and 30% moisture treatments, respectively. To: onset temperature; Tp: peak temperature; Tc: conclusion temperature, Tc–To gelatinization temperature range; ∆H: gelatinization enthalpy.

**Table 3:** Degree of substitution and reaction efficiency of native-OSA and HMT-OSA starches ^†^.

	DS	RE (%)
N-OSA	0.0009 ± 0.0000 ^a^	3.76 ± 0.00 ^a^
H20-OSA	0.0053 ± 0.0001 ^b^	22.06 ± 0.44 ^b^
H25-OSA	0.0086 ± 0.0006 ^c^	35.86 ± 0.25 ^c^
H30-OSA	0.0049 ± 0.0007 ^b^	20.69 ± 1.27 ^b^

^†^ Data are expressed as means ± SD. ^a–c^: Mean values assigned with the same letters within a column are not significantly (*p* < 0.05) different. N-OSA: native sago starch-OSA modification; H20-OSA: HMT 20-OSA modification; H25-OSA: HMT 25-OSA modification; H30-OSA: HMT 30-OSA modification.

**Table 4:** Oil absorption capacity, water solubility, water vapor permeability, and R_1022/995_ ^†^.

	OAC (%)	Water Solubility (%)	WVP (g·s^−1^·m^−1^·Pa^−1^ × 10^−5^)	R_1022/995_
Native	0.65 ± 0.01 ^a^	4.14 ± 0.11 ^a^	6.41 ± 0.55 ^a^	1.17 ± 0.00 ^a^
N-OSA	0.85 ± 0.01 ^b^	9.04 ± 1.07 ^ab^	7.54 ± 0.19 ^a^	1.12 ± 0.00 ^b^
HMT 25	0.78 ± 0.00 ^c^	6.28 ± 1.67 ^b^	7.43 ± 0.14 ^a^	1.07 ± 0.00 ^c^
H25-OSA	1.03 ± 0.03 ^d^	8.44 ± 0.15 ^b^	4.92 ± 0.79 ^b^	1.14 ± 0.00 ^d^

^†^ Data are expressed as means ± SD. ^a–d^: Mean values assigned with the same letters within a column are not significantly (*p* < 0.05) different. N-OSA: native sago starch-OSA modification; HMT25: HMT sago starch treated 25% moisture treatment; H25-OSA: HMT 25-OSA modification.

## Data Availability

The data presented in this study are available on request from the corresponding author.

## Supplementary Materials

The following supporting information can be downloaded at: https://www.mdpi.com/article/10.3390/polym14061086/s1, Table S1: Intensity of X-ray Diffractogram of native and HMT sago starches.
