# Supplementary material for: Dual Modification of Sago Starch via Heat Moisture Treatment and Octenyl Succinylation to Improve Starch Hydrophobicity
Source: Polymers (Basel). 2022 Mar 8;14(6):1086. doi: 10.3390/polym14061086 (PMC8955598; doi:10.3390/polym14061086)
Supplement: Supplementary file 1 [file polymers-14-01086-s001.zip › polymers-1603062-supplementary.pdf]

polymers

MDPI

Article

Dual Modification of Sago Starch via Heat Moisture Treatment and Octenyl Succinylation to Improve Starch Hydrophobicity

Angela Myrra Puspita Dewi [1,2], Umar Santoso [1], Yudi Pranoto [1] and Djagal W. Marseno [1,*]

[1] Department of Food and Agricultural Product Technology, Faculty of Agricultural Technology, Gadjah Mada University, Yogyakarta, 55281 Indonesia; a.puspita@unipa.ac.id (A.M.P.D.); umar_s@ugm.ac.id (U.S.); pranoto@ugm.ac.id (Y.P.)
[2] Department of Agricultural Technology, Faculty of Agricultural Technology, Papua University, Manokwari, 98314 Indonesia
* Correspondence: djagal@ugm.ac.id

Supplementary Data

Table S1. Intensity of X-Ray Diffractogram of native and HMT sago starches.

Sample	Intensity at 2thetha						Crystallinity (%)
	5.57°	15.06°	17.12°	18.07°	23.01°	26.7°	
**Native**	1357±22.63^{a}	3357±13.43^{a}	4036±49.50^{a}	3825±36.77^{ab}	3176±21.21^{ab}	1739±38.18^{a}	36.57±1.20^{a}
HMT 10	1219±16.26^{abc}	3413±14.14^{a}	3976±67.18^{a}	3917±69.30^{a}	3269±19.80^{a}	1709±4.95^{a}	36.74±0.30^{a}
HMT 15	1338±101.1^{ab}	3495±80.61^{a}	4002±4.24^{a}	3892±2.83^{a}	3261±10.61^{a}	1709±16.97^{a}	35.75±2.75^{a}
HMT 20	1119±109.6^{c}	3440±3.54^{a}	3874.5±19.09^{ab}	3893±1.41^{a}	3239±41.01^{ab}	1664±33.94^{a}	32.30±1.65^{ab}
HMT 25	1154±91.92^{bc}	3391±159.09^{a}	3698±61.52^{bc}	3740±36.77^{c}	3033±59.40^{ab}	1719±78.49^{a}	30.16±1.25^{b}
HMT 30	1150±52.33^{bc}	3094±53.03^{a}	3610±149.91^{c}	3634±112.43^{bc}	3009±219.2^{b}	1702±25.46^{a}	28.70±2.76^{b}

† Data are expressed as means ± SD. Mean values with the same letters within a column are not significantly ($p < 0.05$) different. HMT 10: modified HMT sago starch with moisture level 10% treatment; HMT 15: modified HMT sago starch with moisture level 15% treatment; HMT 20: modified HMT sago starch with moisture level 20% treatment; HMT 25: modified HMT sago starch with moisture level 25% treatment; HMT 30: modified HMT sago starch with moisture level 30% treatment.
